# Biodegradation of used engine oil by novel strains of *Ochrobactrum anthropi* HM-1 and *Citrobacter freundii* HM-2 isolated from oil-contaminated soil

**DOI:** 10.1007/s13205-016-0540-5

**Published:** 2016-10-19

**Authors:** Haytham M. M. Ibrahim

**Affiliations:** Radiation Microbiology Department, National Center for Radiation Research and Technology (NCRRT), Atomic Energy Authority, 3 Ahmed El Zomor St., Nasr City, P.O. Box 29, Cairo, 11371 Egypt

**Keywords:** Used engine oil, Biodegradation, *Ochrobactrum anthropi*, *Citrobacter freundii*

## Abstract

Used engine oil (UEO) constitutes a serious environmental problem due to the difficulty of disposal off or reuse. Ten bacterial strains with biodegradation potential were isolated from UEO-contaminated soil sample using enrichment technique. Two strains which exhibited the highest degradation %, 51 ± 1.2 and 48 ± 1.5, respectively, were selected. Based on the morphological, biochemical characteristics and 16S rRNA sequence analysis, they were identified as *Ochrobactrum anthropi* HM-1 (accession no: KR360745) and *Citrobacter freundii* HM-2 (accession no: KR360746). The different conditions which may influence their biodegradation activity, including UEO concentration (1–6 %, v/v), inoculum size (0.5–4 %, v/v), initial pH (6–8), incubation temperature (25–45 °C), and rotation speed (0–200 rpm), were evaluated. The optimum conditions were found to be 2 % UEO, 2 % inoculum size, pH 7.5, incubation temperature 37 °C, and 150 rpm. Under the optimized conditions, strains HM-1, HM-2, and their mixture efficiently degraded UEO, they achieved 65 ± 2.2, 58 ± 2.1, and 80 ± 1.9 %, respectively, after 21 days of incubation. Biodegradation of UEO was confirmed by employing gas chromatography analysis. Gamma radiation (1.5 kGy) enhanced the degradation efficiency of irradiated bacterial mixture (95 ± 2.1 %) as compared to non-irradiated (79 ± 1.6 %). Therefore, strains HM-1 and HM-2 can be employed to develop a cost-effective method for bioremediation of used engine-oil-polluted soil.

## Introduction

Pollution due to petroleum hydrocarbons and its derivatives, including diesel fuel, gasoline, heavy oil, motor oil, fuel residues, and mineral oil, has an increasing influence on the environmental reconquest (Su et al. 2011). They have been recognized as one of the most hazardous wastes (Udeani et al. 2009). Motor oil is a mixture of base lubricant oil and additives, and the base oil contains long-chain (C_16_–C_36_) saturated hydrocarbons and more than 75 % cyclic alkanes (Koma et al. 2003). One billion gallons of waste lubricating oil (also called spent oil) are generated during oil-changing processes from automobile and mechanical workshops, a few of this huge amount is recycled, and most is disposed off by incineration or dumping. Therefore, there is a need to appropriate recycling to avoid their dangerous threat towards the environment (Kalyani and Pandey 2014). Used motor oil contains more metals, toxic, and carcinogenic polycyclic aromatic hydrocarbons (PAHs). Thus, it constitutes a potential threat to humans, animals, and vegetation (Adelowo et al. 2006). Treatment of contaminated soil and water resources takes place using mechanical methods, such as skimming and chemical methods, such as surfactants and dispersants; nevertheless, these methods are costly and with limited efficiency (Huang et al. 2008). In contrast, the utilization of microorganisms to degrade such pollutant has been found to be a promising alternative (Akio et al. 2006). Microorganisms have potential to detoxify hazardous organic compounds by means of polymerization, mineralization, or transformation (Sarma and Sarma 2010). Therefore, as compared to other technologies, microbial degradation (bioremediation) is the method of choice, because it is cost-effective, safe, and environmentally accepted (Singh et al. 2009). Bioremediation process depends on the ability of microorganisms to remove the hydrocarbon pollutants completely (Gómez et al. 2007). Interestingly, numerous hydrocarbon-degrading microorganisms are capable of producing biosurfactants/emulsifiers agents that could increase the solubility of these sparingly or insoluble substrates and facilitate their utilization as sole carbon sources (Franzetti et al. 2010; Su et al. 2011).

Several reports have been established on biodegradation of petroleum hydrocarbons of used engine-oil-contaminated soils. Husaini et al. (2008) had successfully demonstrated the ability of 18 indigenous fungal isolates, isolated from local motor-oil-contaminated areas that were capable of degrading the aliphatic hydrocarbons. *Penicillium* species (P1) completely degrades the *n*-alkanes in the used motor oil after 2 months of incubation. Su et al. (2011) demonstrated the motor-oil-degrading potential of an indigenous *Pseudomonas aeruginosa* SU-1 bacterial strain. This bacterium was found to have the ability to degrade motor oil efficiently upon growing in a medium containing such pollutant as the sole carbon source. Bhattacharya et al. (2015) reported that a newly isolated *Ochrobactrum* sp. C1 could grow with waste lubricants, as the sole carbon and energy source and degrade a wide range of hydrocarbons present in this waste efficiently. Larik et al. (2016) reported the biodegradation of used engine oil and diesel oil using an efficient bacterial consortium A2457: encompassing *Stenotrophomonas maltophilia*, *Bacillus pumilus*, and *B. cereus*. Salam (2016) in his study established the extensive degradation ability of two *Pseudomonas aeruginosa* strains RM1 and SK1 on waste engine oil. He reported the potentials of these strains in the degradation of aromatic, aliphatic, and branched alkane components of waste engine oils. Microbial consortiums have been usually suggested for complete biodegradation of petroleum pollutants (Farahat and El-Gendy 2008), since the constituents of hydrocarbon mixtures differ in their solubility, volatility, and susceptibility to biodegradation, and in contrast, the required sets of enzymes cannot be present in a single microbial strain.

To treat contaminated areas, it is important to employ microorganisms which are indigenous to the contaminated sites and provide excellent degradation potential. Therefore, this work aims to isolate, identify, and characterize native used engine oil utilizing bacterial strains, from UEO-contaminated soil samples, and to estimate the biodegradation potential of the most promising strains, both individually and in combination. In addition, the factors which influence the biodegradation potential of such pollutant were investigated. In an attempt to enhance the biodegradation efficiency of these strains, the effect of gamma radiation was also evaluated.

## Materials and methods

### Collection of soil samples

Three soil samples (each of 100 g), from locations that had heavy spillage of used engine oil, were collected in sterile polyethylene bags; from a depth of 15 cm, after removing the surface layer. The samples were transferred to the laboratory and kept at 4 °C until used for isolation of used engine-oil-degrading bacteria. The soil samples were blackish in color.

### Physicochemical characteristics of soil samples

The pH of the soil samples was determined with a pH meter (3505 Jenway UK). Moisture and total organic contents were determined gravimetrically. Heavy metals composition of the soils was estimated using atomic absorption spectrophotometer following mixed acid digestion and extraction of the soil samples. The total hydrocarbons content of the soil was extracted using *n*-hexane: dichloromethane solvent systems (1:1, v/v) according to the method described by Obayori et al. (2008).

### Used engine oil (UEO)

UEO samples (250 mL) were collected from a local mechanic workshop in sterile 500-mL screw cap glass bottles. Oil samples was transferred to the laboratory and stored in the refrigerator until use. The oil was blackish brown in color. The heavy metals content of UEO was determined using atomic absorption spectrophotometer.

### Medium composition

The base medium used throughout this study was based on the mineral salts medium (MSM) described by Wongsa et al. (2004), and this medium contains the following in g/L: 4.0 NH_4_NO_3_, 4.7 KH_2_PO_4_, 0.119 Na_2_HPO_4_, 0.01 CaCl_2_·2H_2_O, 1.0 MgSO_4_·7H_2_O, 0.01 MnSO_4_·4H_2_O, and 0.015 FeSO_4_·4H_2_O. The MSM was amended with yeast extract (0.005 g/L) to increase the rate of biodegradation. pH was adjusted to 7.0 using 1 N HCl/NaOH prior to sterilization by autoclave at 121 °C for 20 min.

### Isolation of UEO-degrading bacteria

UEO-degrading bacteria were isolated employing the culture enrichment method as follows: Oil-contaminated soil sample (10 g) was added to sterile MSM (100 mL), supplemented with UEO (1 %, v/v) as the sole carbon source, in 250-mL Erlenmeyer flasks, and incubated in a rotary shaker incubator (Gallen Kamp, UK) at 32 °C and 150 rpm for 15 days. Later, 2 mL inoculum was transferred to a fresh MSM and incubated for another cycle. After three successive transfers, 1 mL of the culture was serially diluted in sterile saline solution (9 % NaCl). A 100 μL of the appropriate dilutions were plated onto sterile MSM agar plates previously sprayed with a UEO solution (2 g/20 mL in dichloromethane) and left uncovered in laminar air flow until the solvent has evaporated. The plates were incubated at 32 °C, and the developed bacterial colonies were selected based on their ability to grow and utilize UEO as sole carbon and energy source. Pure colonies on nutrient agar slants were properly labeled, preserved at 4 °C, and subcultured at 4 week interval.

### Oil degradation potential of selected isolates

A single colony of each selected isolate was inoculated into 25 mL nutrient broth medium (Oxoid) in 250-mL flask, and incubated in a shaker incubator (Gallen Kamp, UK) at 32 °C and 150 rpm for 18 h. Following incubation, the cells were separated by centrifugation at 10.000×*g* and 4 °C for 20 min using GL-18B refrigerated centrifuge (USA), and the pellets were washed twice with sterile normal saline solution and resuspended in fresh sterile MSM. One millilitre of the bacterial suspensions was used as an inoculum; to obtain standard inocula, the optical density (O.D_660_) was fixed to 0.5 (Ghazali et al. 2004) using UV–Vis spectrophotometer (Helios Gamma, Thermo Corporation, England), and uninoculated MSM was used as a blank to adjust the spectrophotometer reading to zero. To determine the biodegradation potential, 2 mL of each bacterial suspension was inoculated into MSM (100 mL), supplemented with UEO (2 %, v/v), and incubated as aforementioned for 15 days. The UEO was sterilized separately by filtration. Control experiments (without bacterial biomass and with inactivated biomass by sterilization to assess the adsorption phenomena of substrate to the bacteria) were run simultaneously to assess the abiotic degradation of UEO. Thus, the values of the abiotic controls were subtracted from biotic degradation efficiencies, to report only the degradation caused by living bacterial cells.

## Analytical methods

### Evaluation of bacterial growth

The bacterial growth was evaluated by measuring the optical density (O.D_660_) using UV–Vis spectrophotometer (Helios Gamma, Thermo Corporation, England).

### Total petroleum hydrocarbons (TPH) determination

TPH were determined spectrophotometrically based on the method of Darvishi et al. (2011). Each sample was mixed with an equal volume of hexane, shaken vigorously for 2 min on vortex, and then centrifuged at 10.000×*g* and 4 °C for 15 min. The hexane phases were collected, and the TPH contents were determined by measuring the absorbance at 272 nm using UV–Vis spectrophotometer, and this wavelength was chosen depending on the previous investigation performed using a mixture of UEO/hexane, which showed the highest absorbance at 272 nm. Following the same procedure, a standard curve was prepared using known concentrations of UEO (0.5–5 mg/L). Accordingly, the relationship between the UEO concentration and the absorbance was as follows:

1 $$ Y = 1.0641X $$

where *Y* is the measured absorbance of sample (AB_272nm_) and *X* is the concentration of UEO in the sample.

Degradation percentage was determined as the difference between the initial and final TPH concentrations as follows:

2 $$ {\text{Degradation }}(\% ) \, = \, [(O_{\text{i}} - O_{\text{r}} )/O_{\text{i}} ] \, \times \, 100 \, \% $$

where *O*_i_ is the initial UEO concentration (mg/L) and *O*_r_ is the residual concentration.

### Gas chromatography (GC) analysis

Hexane extracts of residual oil samples were injected into a G-1000 chromatographic analyzer (Dani, Italy) equipped with HP-5 column (30 × 10^3^ cm in length; 0.032 cm in internal diameter; and 1 × 10^−3^ cm in film thickness) and a flame ionisation detector. The operation conditions were sample size (1 μL), nitrogen was used as the carrier gas, the injector and detector temperatures were maintained at 300 °C and 280 °C, respectively, and the oven was programmed at an initial temperature of 40 °C; this was held for 2 min, then ramped at 15 °C/min to 300 °C and held for 10 min. Degradation of UEO was demonstrated by comparing the TPH peaks area of oil residues in inoculated flasks with that from uninoculated control flasks (Bhattacharya and Biswas 2014).

## Screening for biosurfactant production

### Oil displacement assay

Oil displacement test was accomplished, as described by Morikawa et al. (2000). To a Petri plate containing 20 ml of distilled water, a 20 µL of crude petroleum oil was added, forming a thin film on the water surface. Then, 10 µL of cell-free culture supernatants was gently put into the center of oil film. If a biosurfactant is present, the oil will be displaced with an oil-free clearing zone; the diameter of this clearing zone indicates the oil displacement activity of the biosurfactant. Distilled water was employed as the negative control, in which no oil displacement or clear zone was observed. Triton X-100 was used as the positive control.

### Emulsification activity (E_24_)

A mixture of 4 ml culture supernatants and 4 ml used motor oil was mixed by vortex for 3 min and left to stand for 24 h at 25 °C prior to measurement. *E*_24_ was expressed as the percentage of the height of the emulsified layer divided by the total height of the liquid column as given by the following formula (Sarubbo 2006):

3 $$ E_{24}\,(\% ) = \frac{\text{The  height  of  emulsion layer}}{\text{The  height of total solution}}\times 100 $$

### Heavy metal tolerance test

Heavy metal tolerance of strains HM-1 and HM-2 was determined as described by Salam (2016). The strains were grown in nutrient broth overnight at 32 °C and 150 rpm in a rotary shaking incubator (Gallen Kamp, UK). Cells were harvested by centrifugation (5000×*g*; 15 min), washed twice with sterile phosphate buffer, and resuspended in the same buffer solution. The cell concentration of bacterial suspensions was adjusted at 0.8 by measuring the optical density of the samples at 660 nm. Stock solutions (1 M) of metal salts, namely, NiSO_4_, Pb(NO_3_)_2_, and Zn(NO_3_)_2_ were prepared in distilled water, filter sterilized using 0.22-μm membrane filters, and stored in sterile bottles in the dark at 4 °C. Dilutions to 1, 5, 10, and 15 mM of Zn^2+^, Ni^2+^, and Pb^2+^ were made from the stock solutions into nutrient broth. The media were dispensed in 5-ml aliquots and inoculated with 50 μL inoculum. Each of the experiment was conducted in triplicates. Nutrient broth without inocula and heavy metals was used as control. Bacterial growth was measured by absorbance at 660 nm. The maximum tolerance concentration (MTC) for the strains, defined as the highest concentration of metal which do not affect the growth, was determined after incubation for 7 days.

## Identification of bacterial isolates

### Morphological and biochemical properties

The selected bacterial isolates (HM-1 and HM-2) were identified and characterized according to Bergey’s Manual of Determinative Bacteriology (Holt et al. 1998). Morphological characteristics included colony color, Gram staining, spore formation, and motility. In contrast, biochemical tests involved starch hydrolysis, citrate utilization, oxidase production, methyl red test, Voges–Proskauer test, gelatin liquefaction test, fermentation of carbohydrates, production of hydrogen sulfide test, urease test, indole production test, nitrate reduction, and lipid hydrolysis test.

## 16S rRNA sequencing and species identification

Amplification and sequencing of the 16S rRNA gene were performed at Macrogen Biotech Co., Ltd, South Korea. Amplification was performed by polymerase chain reaction (PCR) using DNA Engine Tetrad 2 Peltier Thermal Cycler (BIO-RAD), with the following sets of universal primers 27F (5′-AGAGTTTGATCMTGGCTCAG-3′) and 1492R (5′-TACGGYTACCTTGTTACGACTT-3′), synthesized by Macrogen Biotech Co., Ltd, South Korea. The PCR products were sequenced using ABI PRISM Big Dye Terminator Cycle Sequencing Kit (Applied BioSystems, USA), with the following sets of universal primers: 518F (5′-CCAGCAGCCGCGGTAATACG-3′) and 800R (5′-TACCAGGGTATCTAATCC-3′). Sequencing products were resolved on an Applied Biosystems model 3730XL automated DNA sequencing system (Applied BioSystems, USA). Alignment similarities of 16S rRNA gene sequences, sorted by the E score, were done with BLAST search of the National Centre for Biotechnology Information (NCBI) server (www.ncbi.nlm.nih.gov/BLAST). The 16S rRNA gene sequences were submitted to the GenBank. Analysis of the 16S rRNA gene sequences data was performed using the software package MEGA version 6, and the phylogenetic tree was inferred using neighbor-joining methods.

### Investigation of biodegradation conditions

Oil biodegradation efficiency of strains HM-1 and HM-2 as well as their mixture (with equal volumes) was investigated under various culture and environmental conditions, including the initial UEO concentration (1, 2, 4, and 6 %, v/v), the inoculum size (0.5, 1, 2, and 4 %, v/v), the initial medium pH (6, 7, 7.5, and 8), incubation temperature (25, 32, 37, and 45 °C), and rotation speed (0, 100, 150, and 200 rpm). Under the optimum conditions, the time course of UEO degradation by strains HM-1 and HM-2, and their mixture was determined throughout 21 days. After every 3 days interval of incubation, samples were drawn from each flask for the analysis of bacterial growth and UEO degradation %. All experiments were performed in triplicate.

### Influence of gamma irradiation

Gamma irradiation process was carried out in the National Center for Radiation Research and Technology (NCRRT), using ^60^Co irradiation facility (Gamma Cell 220 Excel, MDS Nordion, Canada). The dose rate was 2.164 kGy/h at the time of experiment. The bacterial strains were inoculated into 50 mL nutrient broth (Oxoid) and incubated for 18 h. Later, aliquots (1 mL) of the culture broth (O.D_660_ = 0.5) were transferred into test tubes containing 9 mL sterile saline solution. The suspensions were then exposed to different doses of gamma radiation (0.5, 1, 1.5, 2, 2.5, and 3 kGy). Afterward, 1 mL of irradiated cells suspensions was inoculated into fresh MSM (100 mL) and enriched with UEO (2 %, v/v). The flasks were incubated as aforementioned. Later, the residual oil was extracted and quantified as mentioned earlier. The efficiency of the irradiated cells was compared with that of the control (non-irradiated) cells.

### Statistical analysis

All experiments were performed in triplicate, and the results are presented as means ± standard deviations. Analysis of variance (ANOVA) was used to compare different variables. All analyses were done using the Statistical Analysis Software (SAS) 9.0.

## Results and discussion

### Physicochemical characteristics of soil samples

The physicochemical properties of the soil samples were performed to determine the physical factors, limiting nutrients, and pollutants that could indicate the types of microorganisms recovered from the soils. The pH of the three soil samples was weakly acidic (5.7–6.3). The presence of various heavy metals, such as lead, zinc, cadmium, iron, and nickel, emphasized the pollution of the soil samples (Table 1).

**Table 1:** Physicochemical characteristics of soil samples

Parameter	Sample 1	Sample 2	Sample 3
pH	6.3	5.7	6.0
Moisture (%)	8.21	8.36	7.56
Total organic carbon (%)	2.01	1.58	1.80
Total hydrocarbon content (mg/kg)	146.12	152.30	188.55
Iron (mg/kg)	21.10	10.53	23.11
Lead (mg/kg)	0.15	0.18	0.21
Zinc (mg/kg)	1.14	0.48	1.20
Copper (mg/kg)	4.20	3.11	2.45
Manganese (mg/kg)	2.25	1.56	3.13
Cadmium (mg/kg)	2.10	1.58	1.20
Nickel (mg/kg)	4.21	2.53	3.33

### Heavy metals analysis of used engine oil

The spectrophotometric analysis of used engine oil demonstrated the presence of several heavy metals, including the following in (mg/l): lead (2.5), nickel (23.4), iron (14), manganese (0.35), copper (0.18), and zinc (55.7).

### Isolation of UEO-degrading bacteria

Employing enrichment technique and plate separation, ten bacterial isolates with apparent clear zones were selected, picked up, and purified. These isolates were designated as HM-1 to HM-10.

### Screening of UEO degrader bacteria

**Fig. 1:**
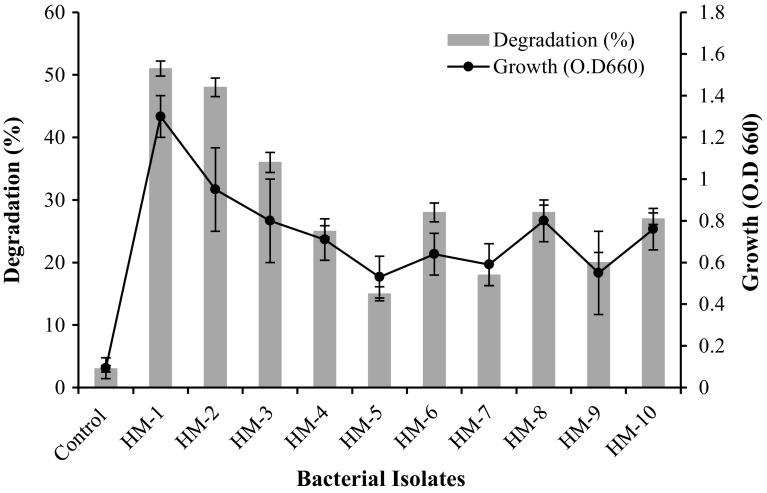
Growth and biodegradation potential of the selected bacterial isolates. *Error bars* represent SD values of three independent experiments (*n* = 3)

As shown in Fig. 1, among the ten bacterial isolates and with degradation potential of more than 45 %, only two isolates, namely, HM-1 and HM-2, efficiently utilized UEO as the sole carbon and energy source, and they showed superior growth; the O.D_660_ was 1.3 ± 0.1 and 0.95 ± 0.2 %, respectively. Besides, they revealed the best degradation efficiency, 51 ± 1.2 and 48 ± 1.5 %, respectively. The other isolates were not further selected and separated because of their low degradability of UEO. Nominal oil degradation (3 ± 0.5 %) was obtained in the abiotic control flasks. During incubation, emulsification of UEO was evident in the culture media, suggesting the production of extracellular biosurfactant/bioemulsifier, which may be one of the mechanisms used by these strains to utilize UEO. Both isolates emulsified the UEO within only 5 days of incubation; on standing the flask, a thin layer of oil was separated out which again became dispersed on gentle shaking. In contrast, the oil layer in control flasks remained on the surface even after 15 days. In a similar study, the strain *Ochrobactrum* sp. C1 was found to tolerate unusually high waste engine oil concentration together with emulsification ability of the culture broth medium, and its degradation competence was 48.5 ± 0.5 % (Bhattacharya et al. 2015).

### Screening for biosurfactant production

Since production of biosurfactant/emulsifier is one of the mechanisms responsible for enhanced biodegradation of water immiscible pollutants, such as UEO, two simple and preliminary tests were employed to determine the ability of strains HM-1 and HM-2 to produce biosurfactant in the culture medium. The cell-free culture supernatants of *O. anthropi* HM-1 and *C. freundii* HM-2 showed high surface activity, and the diameter of displaced circles was 7 and 6.5 cm, with concomitant circles area of 38.5 and 33.17 cm^2^, respectively. Similarly, the cell-free culture supernatants revealed high E_24,_ namely, 90 and 89 %, respectively. Based on these preliminary screening results, it could be stated that strains HM-1 and HM-2 can produce biosurfactants and secrete them extracellularly in the growth medium, and hence, it confirm the speculation of biosurfactant-enhanced biodegradation by these strains. Similarly, Larik et al. (2016) reported the biodegradation of used engine oil and diesel oil under shake flask conditions using an efficient bacterial consortium A2457: comprising bacterial strains of *Stenotrophomonas maltophilia*, *Bacillus cereus,* and *Bacillus pumilus*. They attributed this biodegradation potential to the production of biosurfactants and lipases to utilize diesel oil and used engine oil as sole source of carbon and energy.

### Metal tolerance of used engine-oil-degrading strains

Metal tolerance experiment of strains HM-1 and HM-2 on diverse heavy metals was performed to determine their resistance limit to different concentrations of heavy metals used. The results showed that strains HM-1 and HM-2 resisted zinc, lead, and nickel at (1–15 mM), (1–5 mM), and (1–10 mM), respectively.

### Identification of UEO-degrading bacteria

Following plating on the nutrient agar medium and incubation at 32 °C for 48 h, the developed colonies, of both isolates, were white or creamy-white in color, translucent, convex, with entire margins, smooth surfaces, and 1.5–1.8 mm in diameter. As given in Table 2, the isolates were Gram-negative, non-spore former, rods/short rods, motile, catalase positive, lactose utilization positive, starch hydrolysis positive, and citrate utilization positive. However, there are some differences between the two isolates, as indicated in Table 2. On the basis of the above characteristics, the isolates HM-1 and HM-2 were tentatively identified as *Ochrobactrum* sp. and *Citrobacter* sp., respectively.

**Table 2:** Morphological and biochemical characteristics of the UEO-degrading bacterial isolates

Characteristics	Bacterial isolate	
	HM-1	HM-2
Color of colonies	White	Creamy-white
Shape	Short rods	Rods
Motility	+	+
Gram straining	−	−
Aerobic growth	+	+
Spore formation	−	−
Catalase activity	+	+
Glucose fermentation	−	+
Lactose utilization	+	+
Oxidase test	+	−
Nitrate reduction	−	+
Starch hydrolysis	+	+
Gelatin hydrolysis	−	−
Tween 80 hydrolysis	−	−
Urease hydrolysis	+	−
Indole production	−	−
Methyl red test	−	+
Voges–Proskauer reaction	−	−
Citrate utilization test	+	+
Hydrogen sulfide test	−	−

**Fig. 2:**
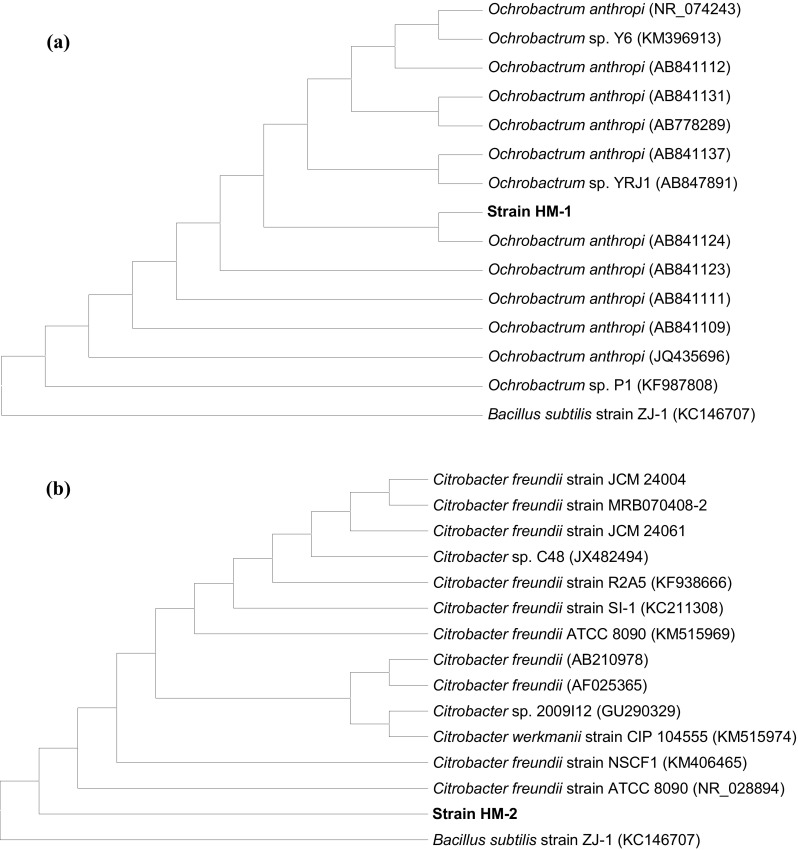
Phylogenetic tree of the 16S rRNA sequences of *O. anthropi* strain HM-1 (**a**) and *C. freundii* strain HM-2 (**b**). The calculations were performed according to a neighbor-joining algorithm (bootstrap number = 1000), and the *scale bar* represents 0.001 sequence divergence. Data in* parentheses* are the GenBank accession numbers

To confirm the results of biochemical and morphological identification, the partial 16S rRNA gene sequences of the two isolates were determined. The 16S rRNA gene sequences of strains HM-1 and HM-2 were a continuous stretch of 996 and 1000 bp, respectively. Database comparison using BLAST program revealed that strains HM-1 and HM-2 had a high similarity of 99 % with those of strains *Ochrobactrum anthropi* (GenBank accession no. AB841124) and *Citrobacter freundii* (GenBank accession no. NR 028894), respectively. Based on the phylogenetic trees, the closest relatives of strains HM-1 and HM-2 were *O. anthropi* (GenBank accession no. AB841124) (Fig. 2a) and *C. freundii* strain ATCC 8090 (GenBank accession no. NR 028894) (Fig. 2b), respectively. Thus, strains HM-1 and HM-2 were designated as *O. anthropi* HM-1 and *C. freundii* HM-2, respectively. The 16S rRNA gene sequences of *O. anthropi* HM-1 and *C. freundii* HM-2 were deposited in the GenBank under accession numbers KR360745 and KR360746, respectively.

### UEO degradation conditions of selected strains

**Fig. 3:**
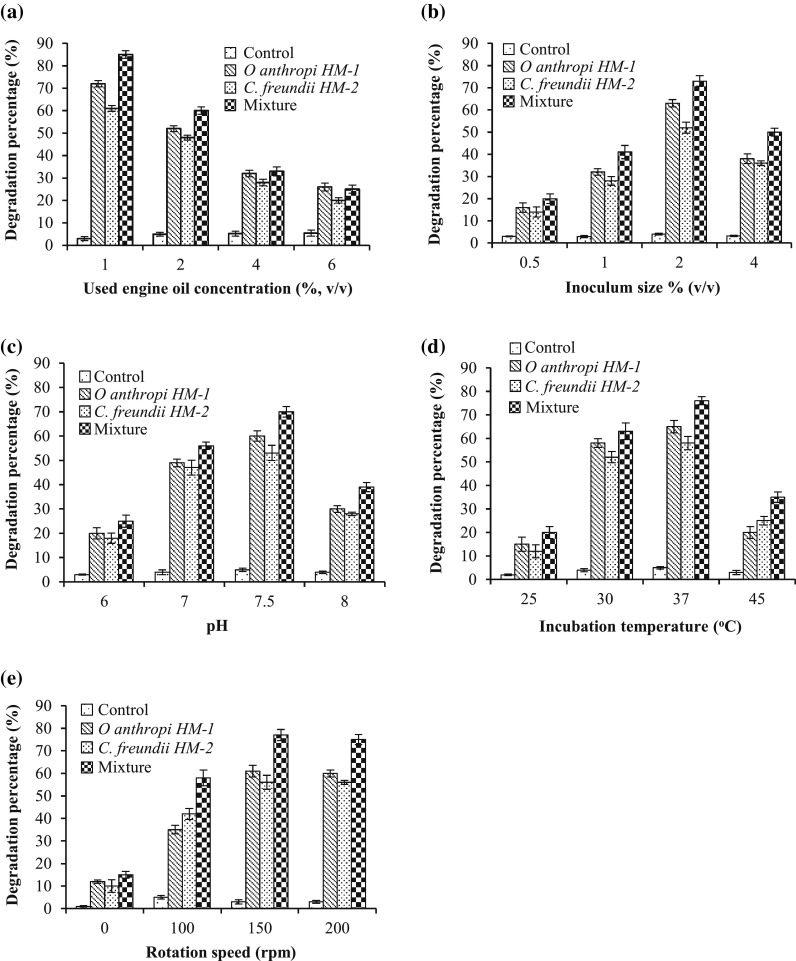
Effect of different cultivation conditions on the biodegradation efficiency of *O. anthropi* HM-1, *C. freundii* HM-2, and their mixture: used engine oil initial concentration (**a**), inoculum size (**b**), medium pH (**c**), incubation temperature (**d**), and rotation speed (**e**). *Error bars* represent SD values of three independent experiments (*n* = 3)

The degradation conditions of UEO were investigated. As illustrated in Fig. 3a, the highest biodegradation percentage was obtained at an initial oil concentration of 1 % (v/v) followed by 2 %. The degradation percentage at 1 % was 72 ± 1.4, 61 ± 1.3 and 85 ± 1.7 %, for *O. anthropi* HM-1, *C. freundii* HM-2, and their mixture, respectively. At higher UEO concentration (4 and 6 %), the degradation percentage decreased significantly, and this decrease could be ascribed to the toxicity of UEO at such concentrations, which might have negative effects on the biodegradation activities of the tested bacterial strains (Abioye et al. 2012). In contrast, for all controls, no significant reduction in oil concentration was observed; the degradation percentage ranged from 3 ± 0.9 to 5.5 ± 1.3 %. For the further investigations, UEO (2 %, v/v) was used. In a similar study, Abioye et al. (2012) reported 3–6 % as the concentration of used motor oil for the maximum rate of biodegradation.

The consumption of nutrients is largely relying on the inoculum size of bacteria; therefore, the bacterial population should be controlled. As illustrated in Fig. 3b, increasing the inoculum size significantly enhanced biodegradation efficiency of tested bacterial strain. The highest degradation percentage was obtained at inoculum size 2 % (v/v). Strains HM-1, HM-2, and their mixture achieved degradation percentage of 63 ± 1.6, 52 ± 2.5, and 73 ± 2.4 %, respectively. Increasing the inoculum size to 4 % resulted in reduced biodegradation percentage. In contrast, no significant reduction in oil concentration was observed for all controls, as pointed out in Fig. 3b. It has been reported that the low inoculum size requires a longer time for cells to multiply and to produce the desired effect (Jin et al. 1998). Similarly, Abusham et al. (2009) mentioned that the small inoculum size can lead to an insufficient number of bacterial cells and a reduced amount of the secreted enzymes, while higher inoculum size could lead to a lack of oxygen and depletion of nutrients in the growth media.

As shown in Fig. 3c, the utilization of UEO was slower when the initial medium pH was acidic (pH 6) as revealed by the low degradation percentage. In contrast, the maximum rate of biodegradation process was achieved when the pH was neutral or slightly alkaline. The highest degradation percentage was observed at pH 7.5, at which *O. anthropi* HM-1, *C. freundii* HM-2, and their mixture attained degradation percentage of 60 ± 2.1, 53 ± 3.1, and 70 ± 2.2 %, respectively. Further increase in medium pH to 8 led to a remarkable decrease in biodegrading percentage, which may be attributed to the reduced bacterial activity. Only marginal abiotic loss (3 ± 0.2 to 5 ± 0.5 %) was observed in the control flasks (Fig. 3c). In good agreement, Bhattacharya et al. (2015) found that the biodegradation of waste lubricants by strain *Ochrobactrum* sp. C1 was optimized at the initial pH 7.3. In addition, Deng et al. (2014) reported that the highest degradation rate (95.6 %) of diesel oil by strain *Achromobacter* sp. HZ01 occurred at pH 7.5. The obtained results were consistent with the findings of Jain et al. (2010) who reported that the degradation of petroleum hydrocarbons in crude oil was most favorable near neutral pH.

Incubation temperature usually varied from microorganism to another, it greatly affects all the metabolic processes, it can be anticipated to have a significant influence on oil degradation, and consequently, it should be controlled. As indicated in Fig. 3d, the rate of UEO degradation process was maximum at incubation temperature of 30 and 37 °C; nevertheless, the most favourable temperature for biodegradation was 37 °C, at which 65 ± 2.6, 58 ± 2.8, and 76 ± 1.7 % degradation were achieved by strains HM-1, HM-2, and their mixture, respectively. A noticeable decreasing trend was observed with the further elevation of incubation temperature to 45 °C. Nominal percentage loss of waste oil was observed for all controls (2 ± 0.3 to 5 ± 0.9 %). In agreement, Aleer et al. (2011) also found that the optimum temperature for waste engine oil biodegradation by a microbial consortium was 30–37 °C.

The variation in the agitation speed affects the degree of mixing and the nutrient availability in the shake flasks or the bioreactor (Abusham et al. 2009). The degradation activity of strains HM-1, HM-2 and their mixture gradually increased and reached the maximum value at rotation speed 150 rpm, at which they exhibited degradation percentage of 61 ± 2.6, 56 ± 3.1, and 77 ± 2.5 %, respectively (Fig. 3e). The degradation rate did not reveal any increasing tendency with the further increase of rotate speed to 200 rpm. Therefore, the optimal rotation speed is 150 rpm, which guarantees to maintain a sufficient supply of dissolved oxygen in the medium for the bacterial strains to grow and degrade the UEO. In agreement, Deng et al. (2014) found that the degradation rate of diesel oil using strain *Achromobacter* sp. HZ01 was gradually increased by increasing the agitation speed and reached the maximum at 150 rpm.

It is worth noted that, for all the degradation experiments, the bacterial mixture exhibited higher degradation percentage compared with the individual pure cultures. These results suggested that strains HM-1 and HM-2 could co-exist with no adverse effect and possibly have a synergy, which may be responsible for the high degradation percentage observed in this study. The advantages of employing mixed cultures have been manifested (Akoachere et al. 2008). Ghazali et al. (2004) reported that some species are able to remove the toxic metabolites that prohibit the activities of the other species. Then, it is possible that the other species degrade complex compounds totally.

### Time course of UEO biodegradation

**Fig. 4:**
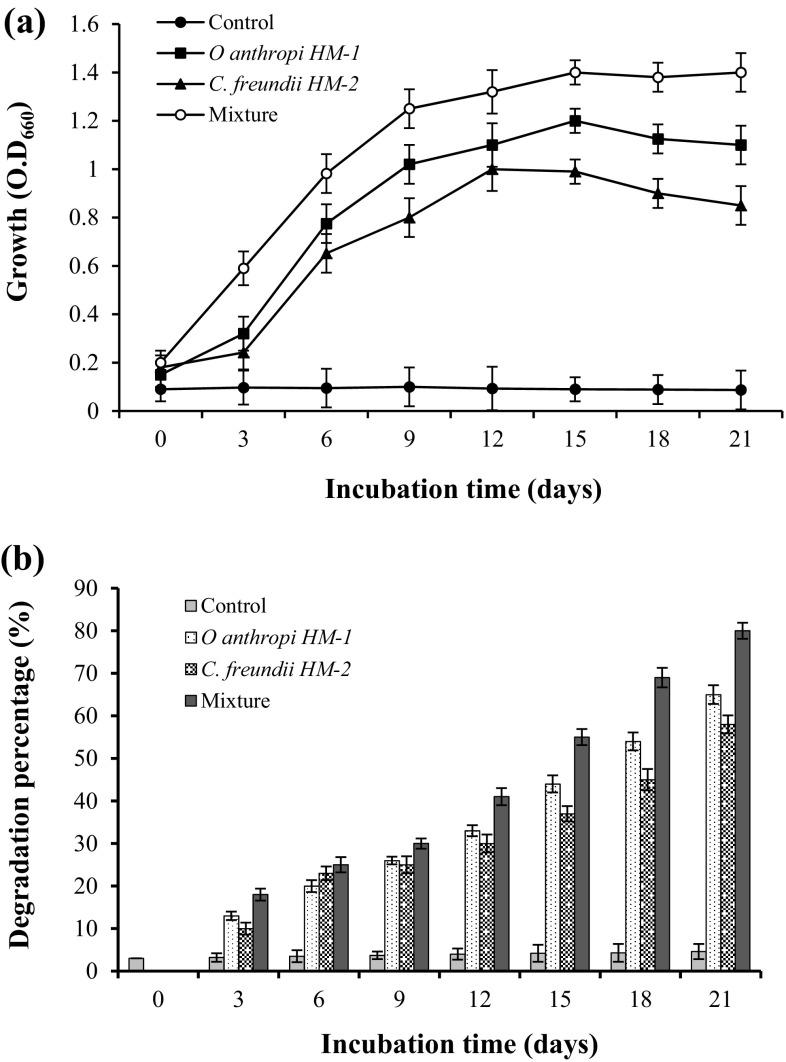
Time course of UEO biodegradation by the bacterial mixture during 21 days of incubation at the initial oil concentration 2 % (v/v), inoculum size 2 % (v/v), medium pH (7.5), incubation temperature 37 °C, and rotation speed 150 rpm

Under the optimum conditions, namely, UEO 2 % (v/v), pH 7.5, inoculum size 2 % (v/v), incubation temperature 37 °C, and 150 rpm, the growth along with degradation rate of UEO by strains HM-1, HM-2, and their mixture were investigated over 21 days incubation period. As illustrated in Fig. 4a, the bacterial strains started utilization of UEO as sole energy and carbon sources, since the initial 3 days interval. The growth (O.D_660_) increased rapidly by extending the incubation time, and strain HM-1 attained maximum growth after 15 days (max. O.D_660_, 1.2 ± 0.05) compared with 12 days for strain HM-2 (max. O.D_660_, 1.0 ± 0.09). In contrast, the bacterial mixture continued to grow and utilize UEO until the end of incubation time, the maximum O.D_660_ was 1.4 ± 0.08. Similarly, the two bacterial strains along with their mixture started degradation of UEO, since the initial 3 days interval (Fig. 4b), and the degradation rate increased with time until reached maximum at the end of incubation period (21 days); 65 ± 2.2, 58 ± 2.1, and 80 ± 1.9 % degradations were attained by strains HM-1, HM-2, and the mixture, respectively. As stated before, the bacterial mixture was more efficient than individual bacterial strains and might be due to synergetic effect.

Larik et al. (2016) reported a significant degradation of the used engine oil (99.77 %) by the consortium A2457 when grown in MSM amended with UEO at 30 °C for 28 days in shaking incubator. Bhattacharya et al. (2015) reported an increase in the percentage degradation from 48.35 to 63.5 % for waste engine oil and from 30.74 to 41.5 % for waste transformer oil on increasing the incubation period up to 21 days. Other researchers (Wang et al. 2011; Abioye et al. 2012) also found that total petroleum hydrocarbon levels could be significantly reduced by increasing the incubation period during treatment of waste oil-contaminated soil.

**Fig. 5:**
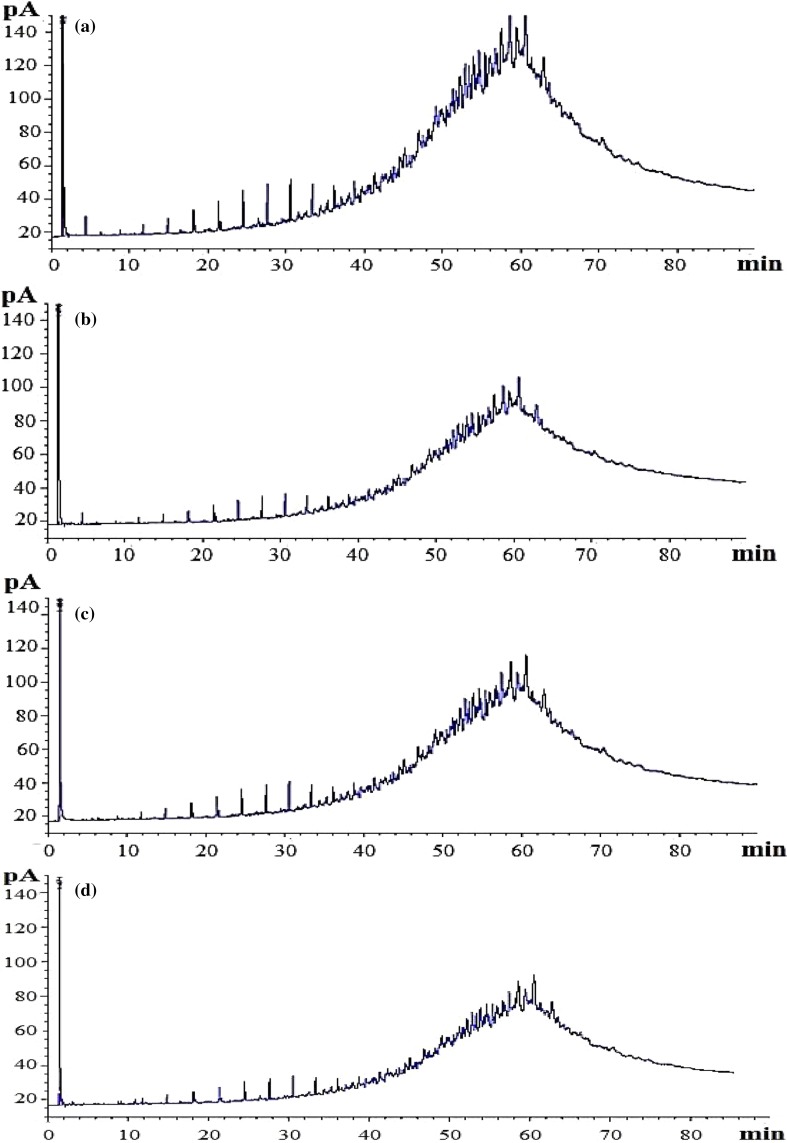
GC chromatograms of residual UEO in MSM after incubation for 21 days at optimum conditions. Abiotic control (**a**), inoculated with strain HM-1 (**b**), strain HM-2 (**c**), and their mixture (**d**)

The GC chromatograms of residual UEO extracted from abiotic control (Fig. 5a), MSM medium inoculated with strain HM-1 (Fig. 5b), strain HM-2 (Fig. 5c), and their mixture (Fig. 5d) exhibited total peak area of, 100,846, 56,307, 75,598, and 50,369, respectively. Thereby, the percentage of total peak area disappeared upon treatments was 44.17, 25.04, and 50.05 %, respectively, as compared to the control. These results confirmed the UEO biodegradation potential of these strains. Besides, the mixture was more efficient than the pure individual cultures. The TPHs were determined in instead of individual petroleum components due to the fact that used engine oil has extremely variable composition and altered structure according to the degree of combustion process during its functioning (Adesodum and Mbagwu 2008).

### Influence of gamma irradiation

**Fig. 6:**
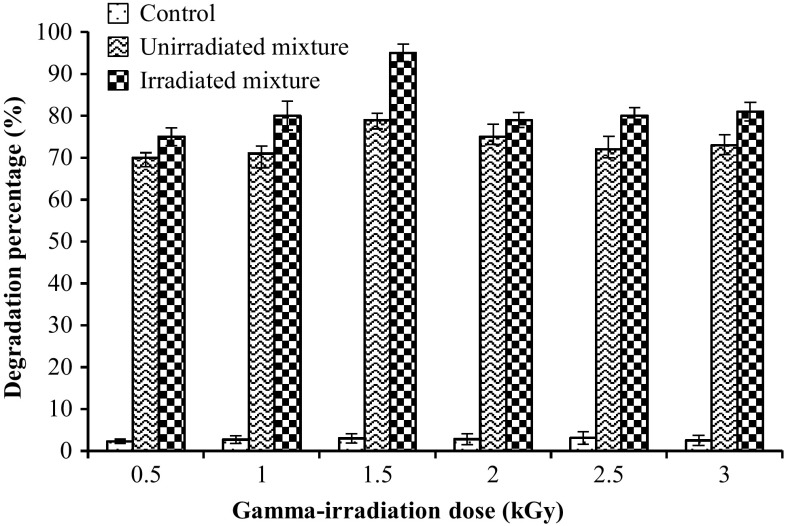
Effect of gamma irradiation on the biodegradation efficiency of *O. anthropi* HM-1 and *C. freundii* HM-2 mixture under optimum conditions. *Error bars* represent SD values of three independent experiments (*n* = 3)

As pointed out in Fig. 6, gamma irradiation enhanced the biodegradation potential of bacterial mixture. The highest biodegradation level (95 ± 2.1 %) was achieved at dose 1.5 kGy as compared to the non-irradiated mixture (79 ± 1.6 %). In contrast, 75, 80, 79, 80, and 81 % were attained at 0.5, 1, 2, 2.5, and 3 kGy, respectively. It is speculated that the improved activity of the irradiated cells may be attributed to the changes occurred at the genetic level, i.e., a mutation in the gene(s) that encode for the enzymes involved in the biodegradation process or the enhancement in biosurfactant/bioemulsifier production which in turn increase the availability of used engine oil. Physical mutagenesis employing different irradiation methods has been adopted as one of many strategies to mutate bacteria. There are many examples concerning the potential efficiency of physical mutagenesis, such as UV and gamma irradiations to enhance the production of different biocatalysts. A gamma-ray-induced mutant of *Pseudomonas aeruginosa* strain S8 was isolated and designated as *P. aeruginosa* EBN-8. This mutant revealed 3–4 times more crude oil biodegradation and emulsification when grown on Khaskheli crude oil in minimal medium as compared to the parent strain (Iqbal et al. 1995). Similarly, an improved biodegradation of crude oil in aqueous phase was achieved utilizing the mutant *Dietzia* sp. obtained by random mutagenesis of parent *Dietzia* sp. employing ^60^Co gamma irradiation. A genetically stable mutant, designated as M22, was isolated and approved significantly higher degradation percentage (52.5 %) of total petroleum hydrocarbons (TPHs) than the wild strain (28.2 %) in liquid media after incubation for 14 days. This enhancement was ascribed to increase production of enzymes responsible for the degradation by the mutant strain (Duan et al. 2015).

## Conclusions

This study demonstrated the potential of native bacterial consortium encompasses *O. anthropi* HM-1 and *C. freundii* HM-2 for bioremediation of UEO-contaminated soil. Each individual strain in the co-culture has a significant role and may be dependent on the presence of the other strain for surviving and biodegradation. The mixture demonstrated higher degradation efficiency of UEO than the individual cultures, possibly due to the synergy effect. Gamma irradiation significantly enhanced the biodegradation potential of the co-culture. Therefore, strains HM-1 and HM-2 can be employed to develop a cost-effective and eco-friendly method for the bioremediation of used engine-oil-polluted soil.
